# Exosome-Enriched Hub Gene Networks Identify Diagnostic Biomarkers and Repurposable Therapeutic Targets in Endometriosis

**DOI:** 10.3390/ijms27062572

**Published:** 2026-03-11

**Authors:** Meng-Hsiu Tsai, Shao-Ping Weng, Li-Jen Su, Tsung-Hsuan Lai

**Affiliations:** 1 Department of Biomedical Science and Engineering, National Central University, Taoyuan City 320317, Taiwan; billy1990914@gmail.com; 2 IHMED Reproductive Center, Taipei City 106028, Taiwan; 3 Education and Research Center for Technology Assisted Substance Abuse Prevention and Management, National Central University, Taoyuan City 320317, Taiwan; 4 Core Facilities for High Throughput Experimental Analysis, Department of Biomedical Science and Engineering, National Central University, Taoyuan City 320317, Taiwan; 5 Department of Obstetrics and Gynecology, Cathay General Hospital, Taipei City 106438, Taiwan; 6 School of Medicine, Fu Jen Catholic University, New Taipei City 242062, Taiwan

**Keywords:** endometriosis, transcriptomics, GEO, differential expression, protein–protein interaction, hub genes, extracellular vesicles/exosomes, liquid biopsy, connectivity mapping, LINCS L1000, drug repurposing

## Abstract

Endometriosis is a heterogeneous chronic inflammatory disorder associated with substantial diagnostic delay and limited therapeutic options, highlighting the need of robust non-invasive biomarkers and actionable molecular targets to complement existing low-sensitivity tests. To identify conserved pathogenic mechanisms with translational potential, here, we uniformly reprocessed three independent the Gene Expression Omnibus (GEO) microarray cohorts (GSE7305, GSE25628, and GSE11691) and applied a strict, directionally consistent intersection strategy to identify conserved transcriptional signals. We identified 262 consensus differentially expressed genes enriched for immunity/inflammation, cell adhesion and migration, and angiogenesis, consistent with key biological hallmarks of lesion establishment and persistence. Protein–protein interaction topology prioritized 11 highly connected hub genes (*VCAM1*, *CCL2*, *MCAM*, *CD14*, *CD24*, *FGFR1*, *SIRPA*, *CSF1R*, *S100A9*, *S100A8*, and *LY96*) that likely act as an integrated immune-adhesion-angiogenesis axis. Notably, 63/262 (24%) of the consensus genes were annotated to the extracellular exosome compartment, supporting their translational relevance as liquid-biopsy candidates. Finally, connectivity mapping using the LINCS L1000 framework nominated small-molecule perturbagens predicted to reverse the endometriosis-associated signature, providing a rational starting point for drug-repurposing experiments. In conclusion, this study elucidates a conserved immune–adhesion–angiogenesis axis driven by an 11-gene hub network in endometriosis. These core regulators represent promising candidates for the development of non-invasive liquid biopsies and precision, non-hormonal therapeutics.

## 1. Introduction

Endometriosis (EM) is a chronic gynecological condition characterized by the presence of endometrium-like tissue outside the uterine cavity [1]. It is estimated to affect 11% of reproductive-aged women worldwide, approximately 200 million individuals, and is identified in up to 50% of patients with chronic pelvic pain or infertility [2,3,4,5,6]. Despite its prevalence, the pathogenesis of endometriosis remains incompletely understood. Several theories have been proposed, among which three are most widely cited: the retrograde menstruation hypothesis, the coelomic metaplasia hypothesis, and the Müllerian remnants hypothesis [2,4,7,8,9]. Sampson’s retrograde menstruation model, the most broadly accepted, posits that endometrial fragments shed during menses reflux through the fallopian tubes into the peritoneal cavity, where they implant and proliferate to form ectopic lesions [1]. Nonetheless, this mechanism alone does not fully account for extra-pelvic or rare gonadal-independent presentations, prompting alternative explanations. The coelomic metaplasia hypothesis suggests that peritoneal mesothelial cells can transdifferentiate into endometrium-like tissue, whereas the Müllerian remnants hypothesis proposes that displaced embryonic Müllerian-derived cells persist and, under specific stimuli such as estrogen exposure or diethylstilbestrol, give rise to ectopic lesions [10].

Current diagnosis typically integrates clinical presentation with imaging and serum biomarkers. Transvaginal ultrasonography and MRI aid in detecting ovarian endometriomas. Although CA-125 is the most commonly used biomarker, its sensitivity and specificity are poor, particularly in early-stage disease. Other markers, such as CA 19-9 or HE4, are primarily used to assist in excluding malignancy rather than for direct diagnosis. Consequently, currently available blood markers lack sufficient accuracy for stand-alone diagnosis [5,7,11]. Hallmark symptoms include chronic pelvic pain, dysmenorrhea, dyspareunia, bowel dysfunction, and infertility [5,7,11]. Some patients also report gastrointestinal complaints (e.g., nausea, vomiting, increased bowel frequency with pelvic pain) and lower urinary tract symptoms, particularly cyclical frequency and urgency [12,13]. Pelvic examination can help localize tenderness, yet definitive diagnosis often relies on invasive methods, underscoring the urgent clinical need to develop novel non-invasive markers with high diagnostic potential.

Disease severity is commonly graded using the revised American Society for Reproductive Medicine (rASRM) system, which stratifies endometriosis into four stages. In Stage I, lesions are few and superficial without notable adhesions; Stage II involves a greater number of predominantly superficial implants with minimal deep disease and no significant adhesions; Stage III features increased lesion burden with the onset of deep infiltration, small ovarian endometriomas (<4 cm), and early pelvic adhesions; and Stage IV denotes extensive disease, large endometriomas (>4 cm), and dense adhesions that may distort pelvic anatomy and tether the ovaries, fallopian tubes, uterus, and bowel (rASRM). Patients with Stage I–II disease may experience limited disruption of daily activities, whereas Stage III–IV disease is frequently associated with severe pain and impaired fertility, leading to a substantial reduction in quality of life.

By anatomic distribution, EM encompasses several phenotypes, including superficial peritoneal lesions, ovarian endometrioma, deep infiltrating endometriosis (DIE), and uterine adenomyosis/adenomyoma. These phenotypes can coexist within the same patient and may reflect partially distinct biological programs [14,15,16,17,18].

Beyond benign morbidity, EM is associated with an increased risk of certain malignancies, most notably ovarian cancer, including endometrioid and clear cell subtypes [19,20]. Although epidemiologic associations are well described, the molecular determinants of malignant transformation remain incompletely defined, reinforcing the value of mechanistic studies that can inform prevention and targeted intervention.

The management of EM is tailored to the severity of the disease and the localization of lesions, utilizing pharmacological, surgical, or combined modalities. The primary goals of medical therapy are to alleviate symptoms and prevent postoperative recurrence [21]. Currently, combined oral contraceptives (COCs) and high-dose progestin regimens are widely employed to suppress ovulation and induce decidualization, thereby reducing lesion size. These therapies are generally well tolerated and cost-effective, providing pain relief for the majority of patients. However, a subset of women may not respond to treatment or may experience adverse effects such as breakthrough bleeding, breast tenderness, nausea, and headaches [22,23]. For patients who are unresponsive to progestin-based therapies, low estrogen regimens serve as an effective second-line alternative. Specifically, gonadotropin-releasing hormone (GnRH) agonists function via a negative feedback loop on the pituitary gland; this inhibits gonadotropin secretion and subsequently downregulates ovarian steroidogenesis. A significant limitation of GnRH agonists, however, is their instability in the digestive tract, which precludes oral administration. Consequently, these agents must be delivered via parenteral, subcutaneous, intramuscular, intranasal, or vaginal routes [24]. Given that current clinical interventions are not universally effective and are often accompanied by adverse effects, there is an imperative need to develop novel therapeutic agents.

Bioinformatics provides the essential computational framework to interpret the massive datasets generated by modern high-throughput technologies, such as next-generation sequencing (NGS) and microarrays [25]. To decipher biological context, the Database for Annotation, Visualization and Integrated Discovery (DAVID; https://davidbioinformatics.nih.gov/, accessed on 8 August 2025) platform facilitates Gene Ontology (GO) and pathway enrichment analysis, linking gene expression to specific biological processes (BP), cellular components (CC), and molecular functions (MF). Following this, protein–protein interaction networks can be constructed via STRING and visualized in Cytoscape, where the cytoHubba plugin allows for the identification of core hub genes based on topological ranking [26]. These analyses are further complemented by databases like Wiki Pathways and the Kyoto Encyclopedia of Genes and Genomes (KEGG) to contextualize signaling alterations. Notably, DAVID’s compatibility with diverse gene identifiers makes it a robust tool for integrating heterogeneous data sources [27]. Beyond elucidating molecular mechanisms, transcriptomic signatures serve as a powerful template for therapeutic discovery. To bridge the gap between gene expression data and clinical translation, the Library of Integrated Network-based Cellular Signatures (LINCS) L1000 dataset provides a comprehensive resource for connectivity mapping. By querying the differentially expressed genes (DEGs) against the L1000 database, it is possible to identify small molecules that induce a gene expression profile inverse to that of the disease state. This “signature reversion” strategy allows for the high-throughput screening of potential therapeutic agents and accelerates the identification of candidates for drug repurposing [28].

In this study, we integrated transcriptomic data from three independent GEO cohorts to derive a robust consensus signature of EM. We then used functional enrichment and network topology to prioritize hub genes with plausible mechanistic roles and translational accessibility, including extracellular vesicle/exosome-associated targets. Finally, we applied the LINCS L1000 connectivity framework to nominate small molecules predicted to reverse the EM signature, providing experimentally testable repurposing hypotheses.

## 2. Results

### 2.1. Data Processing and Quality Control

**Figure 1:**
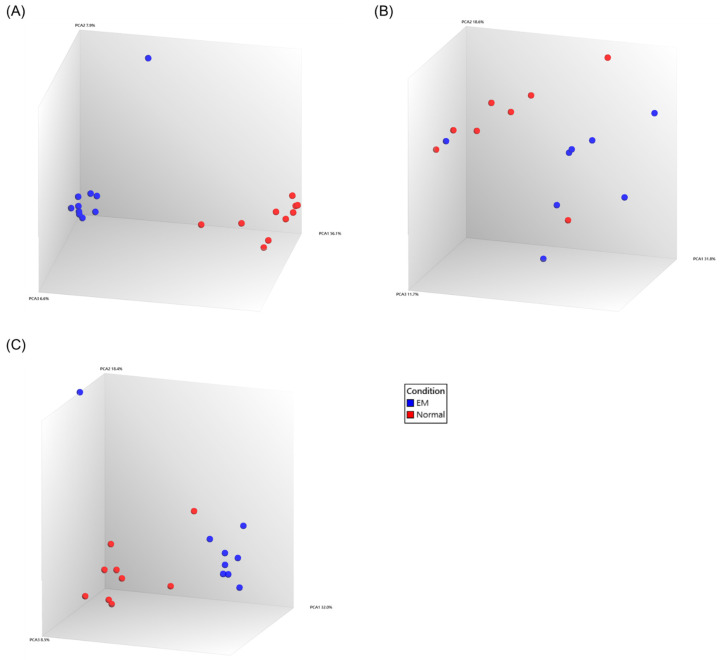
PCA plot of three EM datasets. (**A**) PCA plot of GSE7305. (**B**) PCA plot of GSE25628. (**C**) PCA plot of GSE11691. PCA plots were generated after data preprocessing, which included background correction, quantile normalization, and log_2_ summarization using the Robust Multi-array Average (RMA) algorithm.

Raw Affymetrix microarray CEL files were downloaded from GEO and processed in TAC using a unified workflow. TAC performed automated background correction and quantile normalization, after which we generated PCA plots to assess separation between the EM and Normal groups. Across the three datasets (GSE7305, GSE25628, GSE11691), only GSE7305 displayed clear group separation in the initial PCA (Figure 1A). In contrast, GSE25628 and GSE11691 showed limited separation with partial mixing of EM and Normal samples (Figure 1B,C).

**Figure 2:**
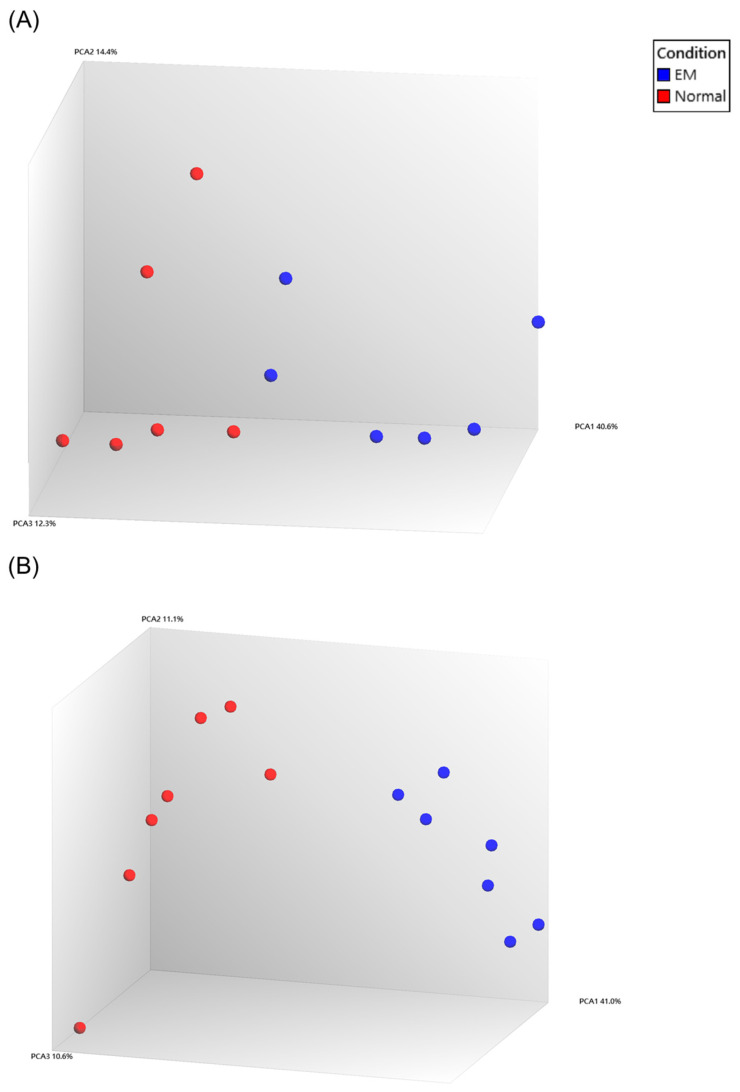
PCA plots after outlier removal for GSE25628 and GSE11691. (**A**) PCA plot of GSE25628. (**B**) PCA plot of GSE11691.

To reduce the influence of outlier samples on normalization and downstream differential expression, we examined sample coordinates along PC1 and removed outlier samples. Because GSE25628 and GSE11691 use paired designs, outliers were removed symmetrically within each pair to preserve pairing. After reprocessing in TAC, PCA plots showed improved clustering in both datasets, with EM and Normal forming two distinct groups (Figure 2A,B).

### 2.2. Differential Expression and Curation

**Figure 3:**
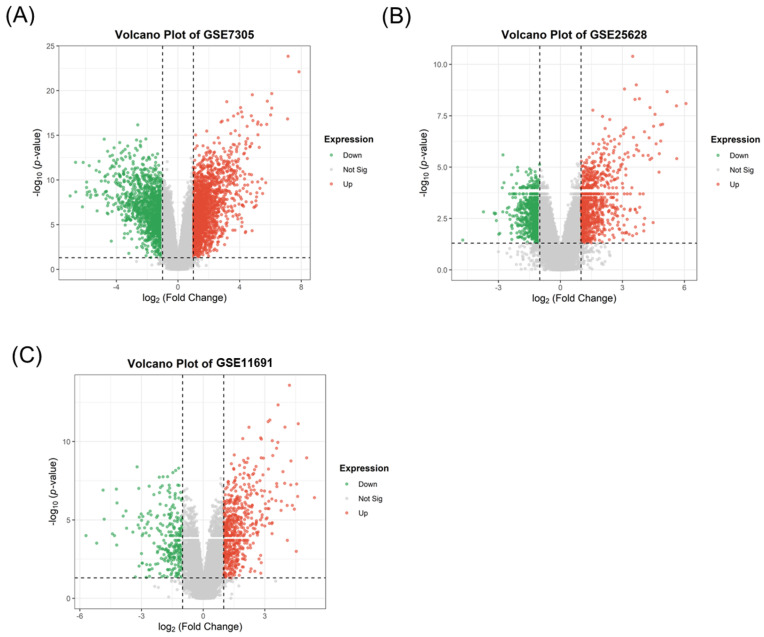
Volcano plots of DEGs comparing EM versus Normal tissue samples. The plots illustrate the significance and magnitude of DEGs identified across (**A**) GSE7305 (EM, *N* = 10; Normal, *N* = 10), (**B**) GSE25628 (EM, *N* = 6; Normal, *N* = 6), and (**C**) GSE11691 (EM, *N* = 7; Normal, *N* = 7). Green dots represent significantly downregulated genes, while red dots represent significantly upregulated genes in EM. DEGs were defined by a strict threshold of nominal *p*-value < 0.05 and absolute fold change ≥ 2.

Following differential expression analysis, the unfiltered numbers of DEGs were: GSE7305, 3966 (2238 upregulated; 1728 downregulated); GSE25628, 1712 (829 up; 883 down); and GSE11691, 859 (581 up; 278 down). We then curated these lists by (i) excluding entries that had a probeset ID but no mapped gene symbol as such records cannot be incorporated into GO analyses, and (ii) collapsing duplicate gene symbols by retaining, for each gene, the entry with the lowest *p*-value. After curation, the final DEG counts were: GSE7305, 2539 (1362 up; 1177 down; Figure 3A); GSE25628, 1417 (675 up; 742 down; Figure 3B); and GSE11691, 693 (465 up; 228 down; Figure 3C). The DEG lists for all three datasets will be provided in Supplementary File S1.

### 2.3. Intersection and Functional Enrichment Analysis

**Figure 4:**
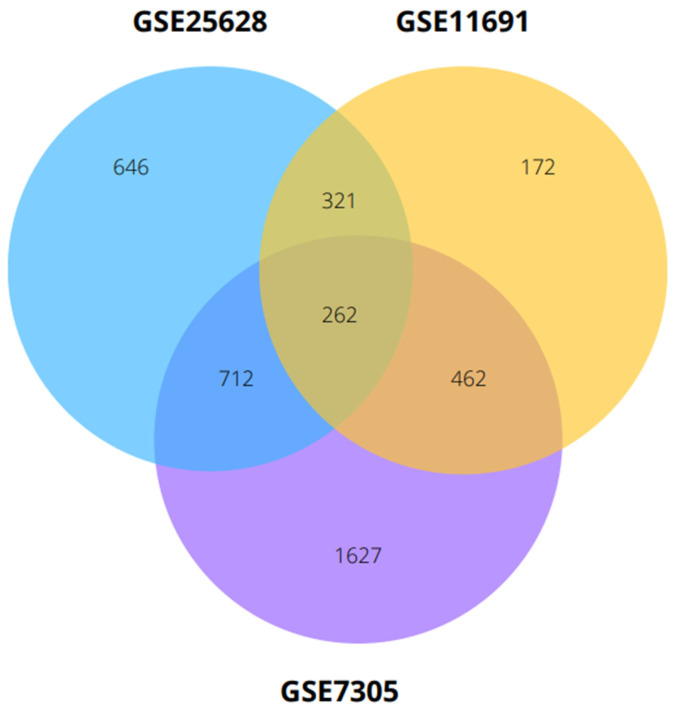
Overlap of DEGs across the three study cohorts. The Venn diagram visualizes the shared and unique significant genes identified in GSE7305 (purple), GSE25628 (blue), and GSE11691 (yellow). The central intersection highlights 262 consensus genes consistently dysregulated across all datasets, which were subsequently used to define the robust molecular signature for network analysis.

To identify a robust molecular signature, we performed an intersection analysis across GSE7305, GSE25628, and GSE11691, requiring directionally consistent log_2_ fold-changes. The overlap structure, including pairwise comparisons and the three-way intersection, is visualized in Figure 4. This consensus approach yielded 262 shared differentially expressed genes (200 upregulated and 62 downregulated; Supplementary File S2).

**Figure 5:**
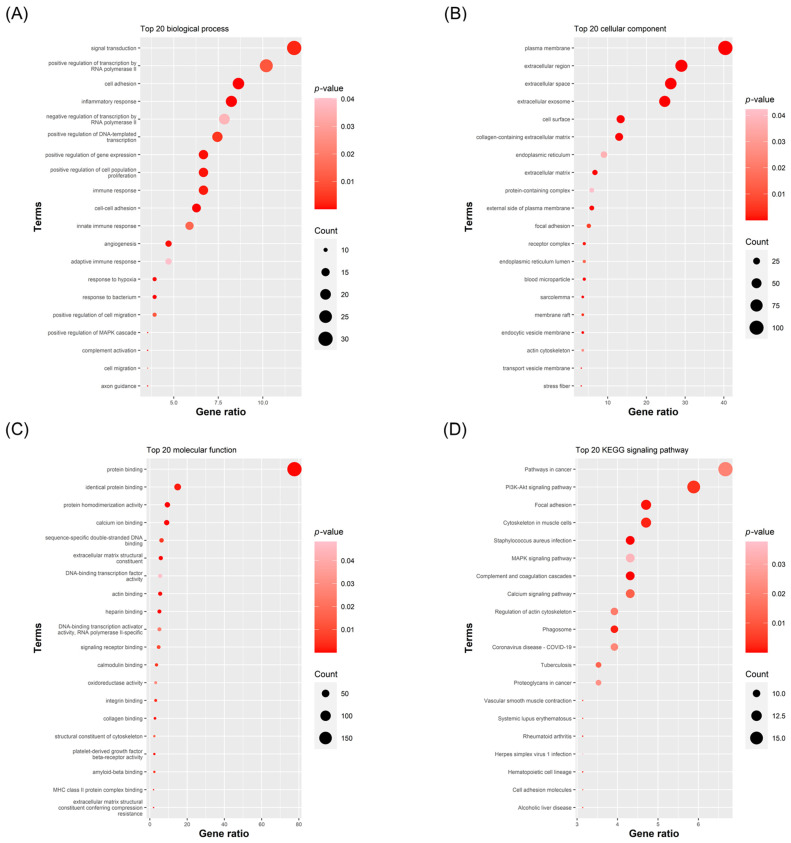
GO and KEGG enrichment of intersecting DEGs from GSE7305, GSE25628, and GSE11691. Bubble plots summarizing the top 20 enriched terms for GO BP (**A**), CC (**B**), MF (**C**), and KEGG pathways (**D**) based on DAVID analysis of the DEGs shared by GSE7305, GSE25628, and GSE11691. The *x*-axis shows the gene ratio. Bubble size encodes the gene count, and color reflects the nominal *p*-value.

Functional enrichment analysis via DAVID revealed that these genes are highly concentrated in biological processes critical to endometriosis pathology. Specifically, GO BP terms were enriched for innate and adaptive immune responses, inflammation, cell adhesion, cell migration, and angiogenesis (Figure 5A). In the Cellular Component category, “extracellular exosome” emerged as a prominent term, consistent with vesicle-mediated communication in lesion biology (Figure 5B). KEGG pathway analysis further corroborated these findings, highlighting immune regulation and matrix dynamics pathways such as PI3K/Akt, MAPK, Complement cascades, and Cell Adhesion Molecules (Figure 5D). The top 20 significant terms were visualized as bubble plots (Figure 5), with full results detailed in Supplementary File S3. Collectively, these functional profiles demonstrate that the 262 consensus genes successfully capture the core pathological landscape of endometriosis spanning immunity, inflammation, and adhesion. This biological coherence provides a robust rationale for using these specific gene sets to construct protein–protein interaction networks and prioritize key regulatory hub genes in the subsequent analysis.

### 2.4. Extracellular Exosome Analysis

Guided by the enrichment results, we focused on the CC term extracellular exosome, which encompassed 63 differentially expressed genes, 24% of the three-way overlap. We cross-referenced these 63 genes against two curated exosome resources, ExoCarta and Vesiclepedia. Of these, 12 genes were listed in both databases, 60 appeared only in ExoCarta, and 3 were absent from both resources. Gene-level annotations and database matches are summarized in Table 1. The high concordance with established exosome databases reinforces the secretory nature of these targets, suggesting that they are likely released into the circulation and could serve as accessible candidates for non-invasive liquid biopsy.

### 2.5. Identification of Pathogenesis-Related Hub Genes via PPI Network Analysis

Guided by the GO BP analysis, we selected highly enriched BP terms corresponding to the core pathophysiological mechanisms that drive the clinical manifestations of endometriosis. Specifically, we focused on innate immune response, adaptive immune response, inflammatory response, angiogenesis, cell migration, and cell adhesion, and retrieved the genes corresponding to each term. For each term-specific gene set, we constructed a PPI network using the STRING database with the organism restricted to Homo sapiens, and imported the resulting interaction tables into Cytoscape. Network prioritization was then performed with the cytoHubba plugin using degree centrality (defined as the number of first-neighbor connections for each node) to identify highly connected candidate hub genes. For each term, nodes were ranked by degree, and the top 7–10 genes were retained; the corresponding core subnetworks were visualized to highlight putative control points within each process.

**Figure 6:**
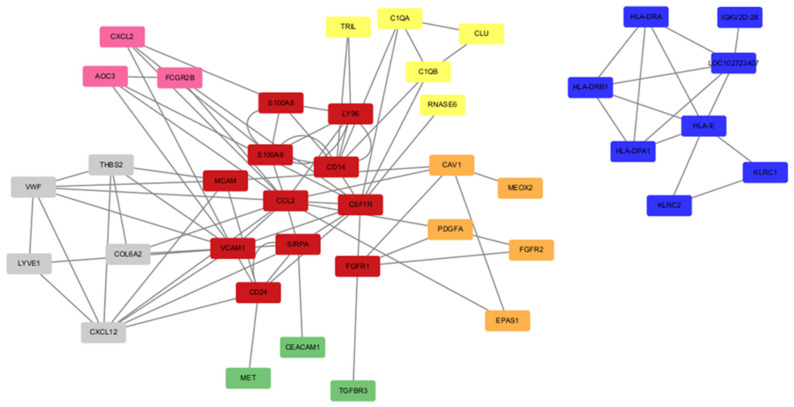
PPI network of BP terms related to common clinical symptoms of EM. The BP terms include innate immune response, adaptive immune response, inflammatory response, angiogenesis, cell migration, and cell adhesion. Node colors indicate the biological processes in which each gene is involved: red, genes participating in ≥2 BP terms; green, cell migration; orange, angiogenesis; yellow, innate immune response; pink, inflammatory response; grey, cell adhesion; blue, adaptive immune response. The network was constructed using the STRING database (Homo sapiens; combined confidence score ≥ 0.40) and visualized in Cytoscape.

This workflow yielded hub networks for innate immune response (yellow), adaptive immune response (blue), inflammatory response (pink), angiogenesis (orange), cell migration (green), and cell adhesion (grey). Several key hub genes (red) participated in two or more BP terms (Figure 6), suggesting that these genes may represent critical regulators underlying BP terms associated with common clinical manifestations of endometriosis. Notably, VCAM1, CCL2, MCAM, CD14, CD24, FGFR1, SIRPA, CSF1R, S100A9, S100A8, and LY96 repeatedly appeared in at least two functional terms. Among these genes, we found that the functional annotations related to common clinical manifestations of endometriosis are tightly interconnected and mutually reinforcing. In addition, VCAM1 is annotated as a component of extracellular vesicles, further enhancing its potential importance in EM.

### 2.6. Small-Molecule Drug Prediction

Guided by the LINCS L1000 framework, we adopted a reverse-signature strategy to nominate compounds predicted to counteract disease-relevant transcriptional changes, i.e., suppress genes commonly up-regulated across the three datasets and/or restore genes commonly down-regulated, thereby potentially exerting therapeutic effects. For each query, L1000 returns a ranked list of candidate perturbagens. Compounds whose mechanisms of action could not be corroborated from the published literature were excluded from further consideration. After curation, the retained compounds clustered into several mechanistic classes. A subset was predicted to modulate immune-related signaling, including WHI-P97 (JAK3 inhibitor), Torin-1 (mTOR inhibitor), and ellipticine (reported as a TLR3 inhibitor). Others primarily targeted proliferation and growth pathways, such as PIK-75 (PI3Kα inhibitor), radicicol (HSP90 inhibitor), and PHA-767491 (CDC7 inhibitor). We also identified BRD-K05151076 as an estrogen-receptor inhibitor. Protein-synthesis inhibitors, including thiostrepton, brefeldin A, and puromycin, were likewise recovered. For each small molecule, we further summarized its perturbed genes and whether those genes participate in endometriosis-related Gene Ontology biological processes (GO BP), including cell adhesion, cell migration, angiogenesis, inflammatory response, innate immune response, and adaptive immune response (details in Table 2).

Notably, we also recovered cerulenin (a fatty-acid synthase inhibitor) and LRRK2-IN-1 (an LRRK2 kinase inhibitor currently investigated in the context of Alzheimer’s disease); both may have additional therapeutic potential for endometriosis. Finally, several predicted small molecules are already approved for clinical use in other indications; their names, approved indications, and regions of approval are summarized in Table 3. As a next step, we plan to evaluate these candidates in cellular and animal models to assess their efficacy for drug repurposing in endometriosis.

### 2.7. Independent in Silico Validation of the Core Hub Network

To orthogonally validate our 11-gene hub network, we analyzed an additional, independent endometriosis microarray dataset (GSE201912). To capture the most distinct pathological signature, we specifically contrasted normal endometrium (controls) against ovarian endometrioma (ectopic lesions). The complete list of differentially expressed genes for this validation cohort is provided in Supplementary File S5. The differential expression status of the 11 hub genes in this validation cohort is summarized in Table 4. Remarkably, 7 out of the 11 hub genes (*CCL2*, *CD14*, *CD24*, *VCAM1*, *CSF1R*, *SIRPA*, and *LY96*) were significantly differentially expressed in this independent test set (*p* < 0.05, the absolute fold change ≥ 2). Importantly, the directionality of expression for all 7 replicated genes was perfectly consistent with our primary multi-cohort consensus signature, reinforcing their robustness as highly conserved pathogenic markers in endometriosis.

## 3. Discussion

In this multi-cohort re-analysis of three independent GEO series, we identified a robust intersection of differentially expressed genes shared across datasets. These genes were significantly enriched in biological processes highly relevant to endometriosis, including innate and adaptive immune responses, inflammation, extracellular matrix adhesion, directed cell migration, and angiogenesis. Network analysis further converged on a set of highly connected nodes, prioritizing *VCAM1*, *CCL2*, *MCAM*, *CD14*, *CD24*, *FGFR1*, *SIRPA*, *CSF1R*, *S100A9*, *S100A8*, and *LY96* as key hubs. Rather than acting in isolation, these genes likely constitute a coherent pathogenic network that sustains the chronic inflammatory and angiogenic microenvironment of ectopic lesions. By bridging immune activation and tissue remodeling, these central mediators contribute to the persistence of clinical symptoms and represent promising candidate targets for future therapeutic intervention.

In our independent in silico validation using dataset GSE201912, 7 out of the 11 hub genes were successfully replicated with consistent directionality when specifically comparing normal endometrium to ovarian endometrioma. This high replication rate reinforces their roles as a robust, highly conserved core signature. Although the remaining 4 hub genes (FGFR1, MCAM, S100A8, and S100A9) were detected but did not strictly meet the stringent differential expression thresholds in this specific validation cohort, likely reflecting platform-specific probe variances or patient-level heterogeneity, substantial experimental evidence firmly supports their pathological relevance. For instance, FGFR1 is significantly overexpressed in ectopic endometrium, where its signaling was correlated with pain symptoms [54]. Similarly, MCAM is a critical cell adhesion molecule that identifies highly angiogenic endometrial mesenchymal stem-like populations [55]. Furthermore, S100A8 and S100A9 function as potent pro-inflammatory damage-associated molecular patterns (DAMPs) secreted by macrophages to facilitate endometriotic vascularization and fibrogenesis [56,57]. Thus, these 11 genes, collectively, remain biologically critical nodes within the endometriosis immune-adhesion-angiogenesis axis.

The enrichment of immune and inflammatory pathways aligns with the well-recognized inflammatory milieu of EM, where dysregulated leukocyte recruitment and cytokine signaling can facilitate lesion survival [58,59,60]. Adhesion and migration terms align with the capacity of shed endometrial fragments to attach to the peritoneum and invade [60,61], while angiogenesis supports lesion survival in ectopic niches [62]. Positioning the identified 11 hub genes spanning chemokines (e.g., *CCL2*), adhesion molecules (e.g., *VCAM1*, *MCAM*), and immune regulators (e.g., *CD14*, *CSF1R*, *S100A8*/*S100A9*) at the convergence of these processes suggests they function as a coordinated pathogenic network. Rather than acting independently, these hubs likely orchestrate the complex interplay between leukocyte-stromal interactions and vesicle-mediated communication that drives lesion microenvironment dysfunction. The repeated appearance of *VCAM1*, *CCL2*, *MCAM*, and innate immune regulators (e.g., *CD14*, *CSF1R*, *S100A8*/*S100A9*) across multiple GO terms supports their role as convergent regulators bridging immune-stromal crosstalk and tissue remodeling.

Our network results generate actionable hypotheses. If this hub network is central to disease pathology, we expect (i) elevated protein levels of these key targets in eutopic/ectopic endometrium and patient plasma (potentially within exosomes) relative to controls; (ii) perturbing their specific signaling axes such as blocking *VCAM1* mediated adhesion or *CCL2*-driven chemotaxis in endometrial stromal/epithelial models will attenuate adhesion/migration readouts; and (iii) the aggregate expression profile of this multigene signature may correlate with pain burden, rASRM stage, or implantation outcomes. While these mechanistic predictions can be evaluated using multiplex IHC/IF, ELISA, and in vitro functional assays, we further prioritize the translational validation of our drug repurposing findings. Specifically, we plan to evaluate the in vivo therapeutic efficacy of the six clinically established compounds listed in Table 3, including HDAC inhibitors (e.g., Vorinostat, Panobinostat) and immunomodulators, using murine models of endometriosis. By assessing their ability to suppress lesion growth and alleviate pain behaviors in a physiological context, we aim to confirm whether these repurposable agents can offer a rapid and effective alternative to current hormonal therapies.

While previous transcriptomic analyses have explored the pathogenesis of endometriosis, the present study introduces several innovative aspects that distinguish it from standard descriptive bioinformatics. First, we employed a strict, directionally consistent intersection strategy across three independent cohorts. Rather than merely pooling datasets, this stringent filtering isolates a highly conserved molecular signature, minimizing cohort-specific or platform-specific artifacts. Second, we uniquely highlighted the translational potential of the exosomal compartment. By revealing that nearly a quarter (24%) of the consensus genes are enriched in extracellular exosomes, our study actively bridges basic transcriptomic discovery with the urgent clinical need for non-invasive liquid biopsies. Finally, we advanced the analysis beyond biomarker identification by integrating the LINCS L1000 connectivity mapping framework. This signature-reversion approach pivots the research toward actionable precision medicine, successfully nominating both FDA-approved agents and novel small molecules as rational candidates for non-hormonal drug repurposing. Together, these integrated multi-omics strategies provide a comprehensive and actionable roadmap from pathogenic network discovery to clinical therapeutic application.

EM has been clinically linked to several conditions, including infertility [63], endometrioid endometrial carcinoma [64], and ovarian clear cell carcinoma [65,66]. With respect to infertility, the association between EM and impaired fecundity is well recognized, yet the underlying mechanisms remain incompletely defined. EM can influence reproduction through multiple, often intersecting pathways: chronic pelvic pain, persistent inflammation, pelvic adhesions, diminished ovarian reserve and function, and reduced endometrial receptivity collectively shape the phenotype of EM-related infertility [63]. Recent reports also highlight the role of uterine natural killer (uNK) cells in implantation [67], and several studies have documented a significant reduction in uNK cells in samples from patients with EM [68]. Together, these observations suggest that EM-associated decreases in uNK abundance may contribute to implantation failure and thus represent one plausible, indirect mechanism linking EM to infertility.

Current management of EM centers on surgical and medical approaches. Surgically, focal to moderate lesions are typically treated by excision or carbon dioxide laser vaporization [69], followed by strategies to prevent postoperative adhesions and their sequelae [70,71,72]. Medically, because EM growth is estrogen-dependent, therapies that counteract endometrial proliferation are prioritized: progestins and oestro-progestins are widely used to alleviate EM-associated pain by suppressing endometrial growth and inducing secretory transformation [69], although a subset of patients exhibits progesterone resistance [4]. Among the predicted compounds, we also identified ER inhibitors. As endometrial proliferation is driven by estrogen stimulation, as discussed above, combining ER inhibitors with current treatment strategies may further enhance therapeutic efficacy. In addition, we plan to experimentally validate the effects of Torin-1, Amuvatinib, LRRK2-IN-1, and other predicted candidates in cellular models of endometriosis to determine whether these agents exert anti-endometriotic activity and could be repurposed as potential adjuvant therapies for EM. Notably, LRRK2-IN-1 has garnered attention beyond its original scope; studies have demonstrated its potent anti-cancer activity against colorectal and pancreatic cancer via inhibition of doublecortin-like kinase 1 [73], as well as its involvement in modulating PINK1/Parkin-dependent mitophagy in the context of Parkinson’s disease [74]. The demonstrated bioactivity of LRRK2-IN-1 across these diverse pathologies suggests it may represent another promising candidate for drug repurposing in endometriosis, warranting further investigation into its specific mechanisms within the endometrial microenvironment. Over the past decade, an expanding literature has also explored adjunctive or alternative modalities, including acupuncture [75], herbal medicine [76], dietary modification [77], physiotherapy, and anti-inflammatory/antioxidant interventions [78] reflecting interest in multimodal, individualized care alongside standard treatments.

Looking forward, we envision the findings of this study translating into clinical practice through two primary translational avenues: non-invasive diagnostics and precision therapeutics. Diagnostically, a major clinical hurdle in endometriosis is the substantial diagnostic delay, which often necessitates invasive laparoscopy. Given that 24% of our consensus signature is significantly enriched in the extracellular exosome compartment, these targets hold substantial promise for the development of blood-based liquid biopsies. In future clinical practice, integrating these exosomal biomarker panels with machine-learning algorithms could allow clinicians to objectively risk-stratify symptomatic patients via a simple blood draw, prioritizing high-risk individuals for early imaging or surgical intervention. Therapeutically, current medical management heavily relies on hormonal suppression, which is unsuitable for patients desiring immediate pregnancy and is often limited by progesterone resistance or adverse effects. Our connectivity mapping rationally nominated several clinically approved compounds, such as HDAC inhibitors and immunomodulators, predicted to reverse the core pathogenic signature. Clinically, we envision these candidates serving as a data-driven foundation for phase II drug-repurposing trials. Ultimately, repurposing these approved agents could provide rapidly deployable, non-hormonal adjuvant therapies for patients who fail standard first-line treatments.

Beyond utilizing signature-reversing compounds identified via LINCS L1000, we also foresee the direct pharmacological targeting of our identified hub genes as a highly viable therapeutic strategy, both currently and in the future. Because our 11-gene hub network constitutes the core of the immune-adhesion-angiogenesis axis, specifically neutralizing these nodes could disrupt lesion survival. For instance, CCL2 is a potent chemokine driving macrophage recruitment. Consistent with similar transcriptomic studies that highlighted the macrophage–chemokine axis in endometriosis, experimental murine models have demonstrated that pharmacological blockade of the CCL2/CCR2 signaling pathway significantly reduces macrophage infiltration and limits ectopic lesion growth [79]. Similarly, targeting angiogenesis via FGFR1 offers another direct approach; several FDA-approved FGFR inhibitors (e.g., Erdafitinib) currently used in oncology could be repurposed and evaluated for endometriosis to starve lesions of their blood supply [80]. Furthermore, future immunomodulatory strategies could specifically target CSF1R or S100A9 to deplete disease-associated macrophages or neutralize pro-inflammatory DAMPs within the pelvic microenvironment [81]. Collectively, these hub-targeted approaches align with emerging literature advocating for precision, non-hormonal immunotherapies [82,83], providing a rational roadmap for transitioning these in silico markers into clinical therapeutic targets.

## 4. Materials and Methods

### 4.1. Data Acquisition

Human endometriosis gene expression datasets were retrieved from the NCBI Gene Expression Omnibus (GEO). To ensure compatibility with our uniform reprocessing pipeline, we specifically selected studies utilizing Affymetrix platforms that provided raw probe-level data (CEL files). The inclusion criteria were defined as follows: (i) samples derived from Homo sapiens, (ii) availability of clear metadata defining case (endometriosis/ectopic) and control (normal/eutopic) groups, and (iii) absence of cohort duplication. Following metadata harmonization and quality screening, three independent datasets, GSE7305, GSE25628, and GSE11691, were selected for downstream analysis.

### 4.2. Microarray Analysis

We processed all raw CEL files in Transcriptome Analysis Console (TAC; Affymetrix/Thermo Fisher, Waltham, MA, USA) following a uniform, pre-specified workflow. Briefly, arrays were imported and assigned to two biological groups EM (endometriosis) and Normal using the series/sample annotations curated from GEO. We performed routine quality control in TAC (array-level signal distributions, boxplots, principal component analysis, and probe-level diagnostics) to screen for technical outliers and artifacts. Specifically, we visualized the first three principal components (PC1, PC2, and PC3) to assess global data structure and sample clustering in three-dimensional space. Expression values were then computed using TAC’s implementation of the Robust Multi-array Average (RMA) procedure, background correction, quantile normalization, and log_2_ summarization to the probeset level, followed by gene-level consolidation using the corresponding platform annotation files. For each dataset, TAC generated a matrix of gene-wise group summaries (mean expression in EM and Normal) and effect sizes (log_2_ fold-change), along with accompanying *p*-values for the EM and Normal contrast. Specifically, genes were considered significantly differentially expressed if they met the threshold criteria of a nominal *p*-value < 0.05 and an absolute fold change ≥ 2, determined via Empirical Bayes ANOVA. The resulting genes by sample matrices and DEG tables were exported for downstream curation and pathway analyses. For orthogonal in silico validation, an independent microarray dataset (GSE201912) was obtained from the GEO database. This dataset was processed and analyzed for differential expression using the identical RMA normalization and statistical thresholds (*p* < 0.05, the absolute fold change ≥ 2) as applied to the primary discovery cohorts.

### 4.3. Bioinformatic Analysis

After generating gene-level summaries of mean expression in the endometriosis group, denoted as EM, and in the Normal group, together with the corresponding fold changes, we harmonized probe annotations and resolved cases in which a single gene was represented by multiple probes. When a gene symbol appeared more than once, we retained the probeset with the smallest *p*-value and removed the remaining entries to avoid inflating downstream counts. Any probeset that lacked a valid gene symbol was excluded to prevent ambiguity in gene-level analyses. We then performed an intersection analysis across the three datasets using Microsoft Access to identify differentially expressed genes shared by all cohorts. The intersecting gene list was submitted to DAVID for GO enrichment across the categories BP, CC, and MF, as well as pathway analysis using the KEGG. For each GO term, we retained the term name, the number of contributing genes, the gene ratio, and the associated *p*-value, and we removed entries with *p*-values greater than 0.05. The remaining terms were sorted in descending order by gene count, and the top twenty were visualized as an enrichment plot generated with the ggplot2 package in R version 4.3.1 [84].

### 4.4. Hub Gene Analysis

Building on the statistically significant results from the standardized GO BP database (*p* < 0.05; Figure 5A), we applied a manual curation step as a secondary biological filter to consolidate highly redundant functional annotations and prioritize processes directly driving the clinical pathophysiology of endometriosis. Specifically, rather than selecting broad, generic cellular functions (e.g., signal transduction) or multiple overlapping parent–child terms, we explicitly focused on highly enriched, distinct representative modules corresponding to the established hallmarks of ectopic lesion development and survival: innate immune response, adaptive immune response, inflammatory response, angiogenesis, cell migration, and cell adhesion. For each selected term, we extracted the corresponding differentially expressed genes using official HGNC gene symbols. We then submitted each term-specific gene list to STRING version 12.0 with the organism set to Homo sapiens to assemble PPI networks, drawing on interaction evidence from curated databases, experimental studies, and coexpression. To limit spurious edges, we required a medium to high confidence threshold, defined as a combined score of at least 0.40. The interaction tables were downloaded in tab-separated (TSV) format and imported into Cytoscape version 3.10.4. Within Cytoscape, we removed isolated nodes, defined as nodes with degree equal to zero, and, where applicable, restricted subsequent analysis to the largest connected component to ensure stable centrality estimates. Network topology was quantified with cytoHubba version 0.1, using Degree as the primary ranking metric, where Degree is the number of first-neighbor connections per node. Specifically, the top 7 to 10 nodes with the highest degree scores within each BP-specific subnetwork were extracted. Finally, a manual curation step was applied to identify intersecting hubs; only those top-ranked genes that concurrently participated in two or more of the selected BP terms were designated as the final core hub genes for downstream analysis.

### 4.5. Prediction of Candidate Small-Molecule Compounds

Candidate small-molecule compounds were predicted using the L1000 platform from the Library of Integrated Network-Based Cellular Signatures (LINCS) [28]. Genes that were commonly deregulated across the three datasets were first identified and then split into upregulated and downregulated gene sets, which were submitted separately to L1000 in “reverse” mode to search for compounds that could oppositely regulate the input signature. For each query, L1000 returned the top 50 ranked small molecules. These candidates were subsequently cross-checked in PubChem to obtain their validated chemical names, and, based on these annotations, each compound was further classified according to its predominant inhibitor type.

## 5. Conclusions

The limited performance of existing non-invasive tests (including CA-125) highlights the need for sensitive and specific biomarkers for endometriosis. By uniformly reanalyzing three independent cohorts, we defined a conserved transcriptional signature and prioritized an 11-gene hub network (*VCAM1*, *CCL2*, *MCAM*, *CD14*, *CD24*, *FGFR1*, *SIRPA*, *CSF1R*, *S100A9*, *S100A8*, and *LY96*) that collectively sustains the inflammatory and angiogenic microenvironment of endometriosis. The enrichment of extracellular vesicle/exosome-associated genes supports the feasibility of translating these targets into liquid-biopsy panels. Connectivity mapping further nominated repurposable compounds predicted to reverse the EM signature, providing a rational, testable starting point for experimental validation in cellular and in vivo models. Moving forward, we plan to integrate laboratory-developed AI algorithms to score and weight these markers, validating their expression in endometrial tissues and circulating exosomes. This multi-modal strategy aims to construct a precise, non-invasive risk-prediction model and accelerate the development of targeted therapies for endometriosis.

Limitations: This study is based on retrospective in silico re-analysis of public microarray datasets; findings require orthogonal validation at the protein level and in independent prospective cohorts. In addition, clinical heterogeneity (lesion type, stage, cycle phase) and platform-specific effects may influence gene expression estimates despite uniform preprocessing.

## Figures and Tables

**Table 1:** Gene list of extracellular exosome.

Presence	Regulatory	Gene Symbol	Gene Description	Fold Change			EM-Related BP Terms
				GSE7305	GSE25628	GSE11691	
Both	Up	*ACTA2*	actin, alpha 2, smooth muscle, aorta	5.9	3.3	3.2	None
		*AEBP1*	AE binding protein 1	14.2	4.2	3.0	None
		*AOX1*	aldehyde oxidase 1	22.9	16.0	3.1	None
		*AQP1*	aquaporin 1 (Colton blood group)	5.0	16.8	7.6	None
		*BGN*	biglycan	5.3	4.2	3.0	None
		*BST2*	bone marrow stromal cell antigen 2	11.0	3.9	3.0	innate immune response
		*C3*	complement component 3	34.6	5.5	3.4	inflammatory response
		*C7*	complement component 7	230.3	67.3	24.7	innate immune response
		*CD14* *	CD14 molecule	3.3	2.1	3.1	innate immune response
		*CD74*	CD74 molecule, major histocompatibility complex, class II invariant chain	2.1	2.2	2.7	None
		*CFB*	complement factor B	4.4	2.8	3.8	None
	Down	*CEACAM1*	carcinoembryonic antigen-related cell adhesion molecule 1 (biliary glycoprotein)	0.2	0.4	0.4	angiogenesis, cell adhesion, cell migration
ExoCarta	Up	*ADIRF*	adipogenesis regulatory factor	17.3	12.9	9.0	None
		*AKR1C1*	aldo-keto reductase family 1, member C1	2.9	5.9	2.2	None
		*CFD*	complement factor D (adipsin)	9.1	8.5	4.1	None
		*CFH*	complement factor H	36.3	6.0	4.4	None
		*CLU*	clusterin	6.5	12.2	8.1	innate immune response
		*COL6A2*	collagen, type VI, alpha 2	4.3	4.4	3.1	cell adhesion
		*CPE*	carboxypeptidase E	5.8	4.8	19.3	None
		*CPVL*	carboxypeptidase, vitellogenic-like	8.9	2.1	4.5	None
		*CRYAB*	crystallin alpha B	3.1	4.2	2.0	None
		*CXCL12*	chemokine (C-X-C motif) ligand 12	3.1	2.0	10.1	cell adhesion
		*DES*	desmin	3.5	19.6	2.4	None
		*FABP4*	fatty acid binding protein 4, adipocyte	12.7	24.8	19.2	None
		*FGL2*	fibrinogen-like 2	5.2	2.4	3.5	None
ExoCarta	Up	*GSN*	gelsolin	2.1	2.5	2.8	None
		*HLA-DRA*	major histocompatibility complex, class II, DR alpha	3.5	2.1	2.3	adaptive immune response
		*HLA-DRB1*	major histocompatibility complex, class II, DR beta 1	3.6	2.4	2.9	adaptive immune response
		*HP*	haptoglobin	3.3	9.0	2.7	None
		*IGKC*	immunoglobulin kappa constant	11.7	8.1	5.9	adaptive immune response
		*IGKV2D-28*	immunoglobulin kappa variable 2D-28	8.6	7.3	2.4	adaptive immune response
		*IGLL5*	immunoglobulin lambda joining 3; immunoglobulin lambda-like polypeptide 5; immunoglobulin lambda variable 3–19	15.5	10.5	6.0	None
		*LAMA4*	laminin, alpha 4	2.5	2.0	5.2	cell adhesion
		*LTBP2*	latent transforming growth factor beta binding protein 2	7.4	2.4	3.5	None
		*LTBP3*	latent transforming growth factor beta binding protein 3	2.4	2.2	2.2	None
		*MAN1C1*	mannosidase, alpha, class 1C, member 1	4.4	2.3	2.3	None
		*MGP*	matrix Gla protein	2.3	3.3	9.3	None
		*MMRN2*	multimerin 2	2.4	5.1	2.6	angiogenesis, cell adhesion
		*MYH11*	myosin, heavy chain 11, smooth muscle	24.7	14.1	4.9	None
		*PLA2G2A*	phospholipase A2, group IIA (platelets, synovial fluid)	45.0	49.5	6.6	inflammatory response
		*PRELP*	proline/arginine-rich end leucine-rich repeat protein	14.4	23.8	4.7	None
		*RARRES1*	retinoic acid receptor responder (tazarotene induced) 1	17.8	3.5	2.1	None
		*S100A10*	S100 calcium-binding protein A10	5.5	3.0	2.4	None
		*S100A8* *	S100 calcium-binding protein A8	2.1	7.6	10.1	inflammatory response, innate immune response
ExoCarta	Up	*S100A9* *	S100 calcium-binding protein A9	2.4	5.0	2.7	inflammatory response, innate immune response
		*SIRPA* *	signal-regulatory protein alpha	3.1	2.3	2.2	cell adhesion, cell migration
		*SLIT2*	slit guidance ligand 2	2.1	2.8	3.2	None
		*TGFBR3*	transforming growth factor beta receptor III	11.0	3.6	2.1	cell migration
		*TNXB*	tenascin XB	3.5	3.9	2.6	cell adhesion
		*VCAM1* *	vascular cell adhesion molecule 1	27.5	9.4	5.14	cell adhesion, inflammatory response
		*VWF*	von Willebrand factor	2.7	2.8	3.53	cell adhesion
	Down	*EPCAM*	epithelial cell adhesion molecule	0.0	0.2	0.1	None
		*GALNT3*	polypeptide N-acetylgalactosaminyltransferase 3	0.4	0.4	0.4	None
		*MME*	membrane metallo-endopeptidase	0.1	0.3	0.1	None
		*MYO6*	myosin VI	0.4	0.4	0.4	None
		*PDIA6*	protein disulfide isomerase family A, member 6	0.3	0.4	0.5	None
		*PLS1*	plastin 1	0.2	0.3	0.4	None
		*RAB25*	RAB25, member RAS oncogene family	0.1	0.4	0.3	None
		*SLC22A5*	solute carrier family 22 (organic cation/carnitine transporter), member 5	0.3	0.5	0.4	None
		*SORD*	sorbitol dehydrogenase	0.1	0.3	0.4	None

* Key hub genes of Protein–protein interaction (PPI).

**Table 2:** Comprehensive profile of L1000-predicted small-molecule candidates, their perturbed genes, and associated biological processes.

Perturbation	Inhibitor Type	Perturbed Genes		GO EM-Related BP Terms Involved
Trichostatin A	Histone deacetylase (HDAC) inhibitor [29]	Up to down	*AEBP1*, *AKR1C2*, *ASPN*, *CELF2*, *CFB*, *COL14A1*, *COL6A2*, *DCLK1, DCN*, *DES*, *DPYSL3*, *FABP4*, *FAM129A*, *GAS1*, *GIMAP6*, *IRAK3, LY96*, *MEF2C*, *MEIS2*, *PDGFA*, *PDGFRL*, *PDLIM3*, *PTGIS*, *RGS5*, *THBS2*, *VWF*	angiogenesis, cell adhesion, inflammatory response, innate immune response
		Down to up	*CD24*, *PLS1*, *TPD52*, *TPD52L1*	cell migration
Thiostrepton	Protein synthesis inhibitor [30], FOXM1 inhibitor [31]	Up to down	*ADH1B*, *C3*, *CALD1*, *CAV1*, *CELF2*, *CSTA*, *CXCL12*, *DCN*, *DES*, *FRY*, *GSN*, *IGFBP5*, *LMCD1*, *LTBP2*, *MAN1C1*, *MEF2C*, *MGP*, *MYH11*, *MYL9*, *PDLIM3*, *PLSCR4*, *PRELP*, *PTGIS*, *RARRES1*, *SVIL*, *THBS2*, *TMEM47*, *TSC22D3*, *TSPAN4*	angiogenesis, cell adhesion, inflammatory response
		Down to up	*CKS2*, *MECOM*	None
BRD-A50737080	ATM/ATR kinase inhibitor [32]	Up to down	*DH1B*, *ASPN*, *C7*, *CALD1*, *CCL2*, *CRYAB*, *CXCL12*, *DCN*, *FILIP1L*, *FRY*, *GSN*, *IGFBP5*, *LTBP2*, *MEF2C*, *MGP*, *MN1*, *MYH11*, *PDGFA*, *PLA2G2A*, *PLN*, *PRELP*, *PTGIS*, *RGS2*, *TGFBR3*, *THBS2*, *TSPAN4*, *WISP2*	angiogenesis, cell adhesion, cell migration, inflammatory response, innate immune response
		Down to up	None	None
Brefeldin A	protein synthesis inhibitor [33]	Up to down	*ADH1B*, *ADM*, *AGTR1*, *AKR1C2*, *AOC3*, *AOX1*, *C1QA*, *C1QB*, *C7*, *CAV1*, *CFD*, *CSTA*, *CXCL12*, *DCN*, *FABP4*, *GEM*, *GHR*, *IGFBP6*, *KLF2*, *MEIS2*, *PCOLCE2*, *RGS2*, *S100A10*, *S100A8*, *TAGLN*, *TPSAB1*, *TSPAN4*	angiogenesis, cell adhesion, cell migration, inflammatory response, innate immune response
		Down to up	*TPD52*, *TPD52L1*	None
Vorinostat	HDAC inhibitor [34]	Up to down	*ACACB*, *ADH1B*, *AEBP1*, *AKR1C1*, *AKR1C2*, *ASPN*, *COL6A2*, *CRYAB*, *DPYSL3*, *ECM2*, *FABP4*, *FAM129A*, *FZD7*, *GHR*, *IGFBP6*, *IGKC*, *IRAK3*, *NRN1*, *PCOLCE2*, *PTGIS*, *RARRES1*, *RGS5*, *SORBS1*, *SYNM*, *TNS1*, *VWF*	adaptive immune response, cell adhesion, cell migration
		Down to up	*CD24*, *PLS1*, *SLC39A6*, *TPD52*	cell adhesion
Whi-P97	JAK3 inhibitor [35]	Up to down	*AEBP1*, *AOX1*, *BGN*, *CD74*, *CFD*, *CFH*, *CPE*, *DPYSL3*, *FGL2*, *HLA-DPA1*, *HLA-DPB1*, *HLA-DRA*, *HLA-DRB1*, *IGKC*, *MATN2*, *MYH11*, *PDGFD*, *PDGFRL*, *PLA2G2A*, *PTGIS*, *RCAN2*, *RGS5*, *THBS2*, *TMEM176B*	adaptive immune response, cell adhesion, inflammatory response
		Down to up	None	None
Torin-1	mTOR inhibitor [36]	Up to down	*ADH1B*, *AKR1C1*, *AKR1C2*, *C3*, *CCL2*, *CPE*, *CSTA*, *DMD*, *FABP4*, *FGL2*, *FRZB*, *FXYD1*, *IGKC*, *ITM2A*, *MFAP4*, *MGP*, *RCAN2*, *RGS5, SLIT2*, *SORBS1*, *TMEM47*, *TPSAB1*, *TPSB2*	adaptive immune response, angiogenesis, cell adhesion, inflammatory response
		Down to up	*FGFR2*, *MAP7*, *MYO6*, *SH3YL1*	angiogenesis
Amuvatinib	Multi-target RTK inhibitor [37]	Up to down	*ABCA8*, *ADH1B*, *CALD1*, *CCL2*, *CFB*, *CLU*, *CPE*, *DCN*, *DPYSL3*, *FAM129A*, *FZD7*, *HSD17B6*, *MATN2*, *MEIS2*, *MGP*, *MYH11*, *PLA2G2A*, *PLN*, *RNASE1*, *SORBS1*, *TGFBR3*, *VCAM1*	angiogenesis, cell adhesion, cell migration, inflammatory response, innate immune response
		Down to up	*PTP4A1*	None
OSI-930	multi-targeted RTK inhibitor [37]	Up to down	*AQP1*, *ASPN, BGN*, *CALD1*, *CCL2*, *CPE*, *DCN*, *DPYSL3*, *EPAS1*, *FAM129A*, *FILIP1L*, *GSN*, *ITM2A*, *MEIS2*, *MGP*, *MYH11*, *PLN*, *PRELP*, *RGS5*, *SORBS1*, *THBS2*, *TMEM47*, *TNS1*	angiogenesis, cell adhesion, inflammatory response
		Down to up	*FGFR2*	angiogenesis
Pik-75	PI3Kα inhibitor [38]	Up to down	*ABCA8*, *C7*, *CALD1*, *CD74*, *CDKN1C*, *CFD*, *CFH*, *CSGALNACT1*, *CXCL12*, *DCN*, *FZD7*, *HLA-DPA1*, *HLA-DPB1*, *HLA-DRA*, *HOXC6*, *IGFBP5*, *LAMA4*, *MCAM*, *MEOX2*, *MGP*, *NRN1*, *PCOLCE2*, *RARRES1*, *SLIT2*, *SVIL*, *SYNM*, *TGFBR3*	adaptive immune response, angiogenesis, cell adhesion, cell migration, innate immune response
		Down to up	*ESR1*, *MME*, *PTP4A1*	None
Cerulenin	Fatty acid synthase (FASN) inhibitor [39]	Up to down	*ABCA8*, *BST2*, *C3*, *C7*, *CALD1*, *CAV1*, *CD74*, *CDKN1C*, *COL6A2*, *CSGALNACT1*, *FABP4*, *HLA-DPA1*, *HLA-DPB1*, *HLA-DRA*, *HLA-DRB1*, *LAMA4*, *LHFP*, *MATN2*, *MCAM*, *MGP*, *MYH11*, *NFIB*, *NRN1*, *RGS5*, *S100A10*, *TMEM47*	adaptive immune response, angiogenesis, cell adhesion, inflammatory response, innate immune response
		Down to up	*MME*	None
Panobinostat	HDAC inhibitor [40]	Up to down	*ACACB*, *ADH1B*, *ADM*, *AKR1C1*, *AKR1C2*, *AOC3*, *ASPN*, *COL6A2*, *DPYSL3*, *FABP4*, *FAM129A*, *FMOD*, *GAS1*, *GHR*, *HOXC6*, *LAMA4*, *LPP*, *NRN1*, *PCOLCE2*, *PDGFRL*, *PTGER3*, *PTGIS*, *PTRF*, *SORBS1*, *THBS2*	cell adhesion, inflammatory response
		Down to up	*CD24*, *PLS1*, *TPD52*	cell adhesion, cell migration
Ellipticine	Toll-like receptor 3 (TLR3) inhibitor [41]	Up to down	*AEBP1*, *BGN*, *CALD1*, *CRYAB*, *DCN*, *FAM129A*, *IGFBP5*, *IGKC*, *KLF2*, *LTBP2*, *MEF2C*, *MGP*, *MYL9*, *PCOLCE2*, *PDLIM3*, *PLTP*, *PRELP*, *PTGIS*, *RGS2*, *VCAM1*, *ZFPM2*	adaptive immune response, angiogenesis, cell adhesion, inflammatory response
		Down to up	*ACSL5*, *GALNT3*	None
Amsacrine	Topoisomerase II inhibitor [42]	Up to down	*ACACB*, *ADH1B*, *AKR1C1*, *AKR1C2*, *AQP1*, *BST2*, *CRYAB*, *CXCL12*, *DCN*, *EGR1*, *FMO2*, *GAS1*, *GHR*, *GPC3*, *KLF2*, *MGP*, *PCOLCE2*, *PTGIS*, *SYNM*, *THBS2*, *TSC22D3*, *VCAM1*	cell adhesion, cell migration, inflammatory response, innate immune response
		Down to up	*PTP4A1*	None
Zlllal	Proteasome inhibitor [43]	Up to down	*ACTA2*, *CAV1*, *CRYAB*, *CXCL12*, *DCN*, *DES*, *FABP4*, *FEZ1, GAS1*, *GSN*, *IGFBP6*, *LHFP*, *LTBP2*, *MGP*, *NRN1*, *PRELP*, *PTGIS*, *PTRF*, *SYNM*, *THBS2*, *TMEM47*, *TSPAN4*	angiogenesis, cell adhesion, cell migration
		Down to up	*GALNT3*, *GZMA*	None
Radicicol	Hsp90 inhibitor [44]	Up to down	*ASPN*, *C3*, *C7*, *CCL2*, *CFD*, *COL14A1*, *COL6A2*, *CXCL12*, *DPYSL3*, *EGR1*, *GATA6*, *IGKC*, *KLF2*, *LMCD1*, *MFAP4*, *THBS2*, *TPSAB1*, *TSPAN7*, *VCAM1*	adaptive immune response, angiogenesis, cell adhesion, inflammatory response, innate immune response
		Down to up	*CEACAM1*, *FGFR2*, *GRAMD1C*, *MAP7*, *MYO6*	angiogenesis, cell adhesion, cell migration
PHA-767491	CDC7 dual inhibitor [45]	Up to down	*AEBP1*, *ASPN*, *BGN*, *C7*, *CSGALNACT1*, *DCN*, *DPYSL3*, *FAM129A*, *FILIP1L*, *IGFBP5*, *KLF2, LTBP2, MGP*, *MYH11*, *NRN1*, *PTGIS*, *RARRES1*, *RNASE1*, *S100A8*, *S100A9*, *SLIT2*, *SYNM*, *VWF*	cell adhesion, inflammatory response, innate immune response
		Down to up	None	None
QL47	Covalent BTK tyrosine kinase inhibitor [46]	Up to down	*ACTA2*, *AEBP1*, *AQP1*, *BGN*, *C7*, *CALD1*, *CAV1*, *CFH*, *CSTA*, *CXCL12*, *DCN*, *MAN1C1*, *MCAM*, *MGP*, *PLTP*, *PTGIS*, *RGS2*, *RGS5*, *THBS2*, *TSC22D3*, *VCAM1*	angiogenesis, cell adhesion, inflammatory response, innate immune response
		Down to up	*CD24*, *EPCAM*, *GMFB*, *RBM47*	cell adhesion, cell migration
BI-2536	PLK1/BRD4 dual inhibitor [47]	Up to down	*ADH1B*, *ASPN*, *BST2*, *CCL2*, *CFH*, *CLU*, *COL14A1*, *COL6A2*, *COL8A2*, *CPE*, *DCN*, *FAM129A*, *GAS1*, *GNG11*, *IGKC*, *ITM2A*, *MGP*, *NFIB*, *PDGFRL*, *PTGIS*, *RARRES2*, *RGS5*, *S100A9*, *TPSAB1*, *TSPAN4*	adaptive immune response, angiogenesis, cell adhesion, inflammatory response, innate immune response
		Down to up	*PAPSS1*	None
Celastrol	YAP1 inhibitor [48]	Up to down	*ADH1B*, *ASPN*, *BST2*, *C3*, *C7*, *CAV1*, *CCL2*, *CFB*, *CPE, CXCL12*, *DCN*, *FABP4*, *FRZB*, *GNG11*, *IGFBP5*, *IGKC*, *MCAM*, *MGP*, *NBL1*, *NFIB*, *PLN*, *RARRES1*	adaptive immune response, angiogenesis, cell adhesion, inflammatory response, innate immune response
		Down to up	None	None
Chelidonine	STK19 inhibitor [49]	Up to down	*ABCA8*, *ACACB*, *ACTA2*, *ADH1B*, *AKR1C1*, *AKR1C2*, *CFD*, *CHL1*, *DCN*, *FZD7*, *HLA-DPB1*, *HLA-DRA*, *HOXC6*, *IGFBP5*, *LMCD1*, *MGP*, *S100A10*, *SORBS1*, *THBS2*, *VCAM1*	adaptive immune response, cell adhesion, inflammatory response
		Down to up	*MME*	None
Ouabain	Na+/CK+ ATPase inhibitor [50]	Up to down	*ADH1B*, *C7*, *DES*, *DPYSL3*, *FAM129A*, *GHR*, *HLA-DPA1*, *IGFBP5*, *MGP*, *MICAL2*, *MYH11*, *NFASC*, *PCOLCE2*, *PLN*, *PTGIS*, *RARRES2*, *SORBS1*, *SYNM*, *THBS2*, *ZFPM2*	adaptive immune response, cell adhesion, inflammatory response, innate immune response
		Down to up	*CKS2*, *ESR1*, *SCGB2A1*	None
BRD-K05151076	Estrogen receptor (ESR1/ESR2) inhibitor [51]	Up to down	*ADH1B*, *C7*, *CALD1*, *CRYAB*, *DCN*, *FRY*, *GAS1*, *IGFBP5*, *MEF2C*, *MGP*, *MN1*, *NRN1*, *PDLIM3*, *PTGIS*, *RGS2*, *S100A8*, *S100A9*, *TGFBR3*, *TMEM47*, *TPSAB1*	angiogenesis, cell migration, inflammatory response, innate immune response
		Down to up	*CD24*, *PDIA6*	cell adhesion, cell migration
Puromycin	Protein synthesis inhibitor [52]	Up to down	*ACTA2*, *AGTR1*, *CAV1*, *CELF2*, *CRYAB*, *CXCL12*, *DCLK1*, *DES*, *DPYSL3*, *GAS1*, *GSN*, *LMCD1*, *LTBP2, MEF2C*, *PDLIM3*, *PLN*, *RGS2*, *THBS2*, *TMEM47*	angiogenesis, cell adhesion, inflammatory response
		Down to up	*GALNT3*, *HMGCR*, *PLS1*	None
Lrrk2-IN-1	Type I ATP-competitive LRRK2 kinase inhibitor [53]	Up to down	*ADH1B*, *AEBP1*, *ASPN*, *BGN*, *C3*, *CCL2*, *CFB*, *COL14A1*, *COL6A2*, *CPE*, *CXCL12*, *DCN*, *FABP4*, *FAM129A*, *MYH11*, *NBL1*, *PLN*, *RARRES2*, *SORBS1*, *TPSAB1*, *TPSB2*, *TSPAN4*	angiogenesis, cell adhesion, inflammatory response, innate immune response
		Down to up	*PLS1*	None

**Table 3:** Clinical approvals of selected drugs: approved indications and regions.

Drug Name	Clinical Status	Primary Indication	Approval/Primary Region
Vorinostat	Approved	Cutaneous T-cell Lymphoma (CTCL)	USA (FDA), Japan, EU (EMA), Taiwan and others
Panobinostat	Approved	Multiple Myeloma	EU (EMA)
Amsacrine	Approved	Acute Lymphoblastic/Non-lymphoblastic Leukemia	Europe (select countries), New Zealand, Asia (Not FDA approved)
Ouabain	Restricted/Historical	Heart Failure/Arrhythmia	Germany (Homeopathy), Historical use in EU (Rarely used in modern medicine)
Thiostrepton	Veterinary Use	Dermatological infections in animals	USA (FDA Veterinary), Global (Veterinary only)
Celastrol	Traditional Med/Clinical Trials	Anti-inflammatory/Obesity (Trials)	China (Herbal component), Global (Pure compound in trials)

FDA, U.S. Food and Drug Administration; EMA, European Medicines Agency.

**Table 4:** Independent in silico validation of the 11 core hub genes in dataset GSE201912 comparing normal endometrium versus ovarian endometrioma.

Gene Symbol	Regulatory	Fold Change in GSE201912	*p*-Value in GSE201912	Validated
CCL2	Up	25.64	0.0015	Yes
CD14	Up	4.26	0.001	Yes
CD24	Down	−2.26	0.0094	Yes
CSF1R	Up	4.18	0.0002	Yes
LY96	Up	6.3	0.0002	Yes
SIRPA	Up	2.65	0.0064	Yes
VCAM1	Up	41.75	0.0026	Yes
FGFR1	Up	1.87	0.0205	No
MCAM	Up	1.6	0.0082	No
S100A8	Up	1.01	0.9855	No
S100A9	Up	1.08	0.9623	No

## Data Availability

The microarray datasets analyzed in this study are publicly available from NCBI GEO (GSE7305, GSE25628, and GSE11691). Curated DEG tables and enrichment outputs are provided as Supplementary Files.

## Supplementary Materials

The following supporting information can be downloaded at: https://www.mdpi.com/article/10.3390/ijms27062572/s1.
