# Supplementary material for: Exosome-Enriched Hub Gene Networks Identify Diagnostic Biomarkers and Repurposable Therapeutic Targets in Endometriosis
Source: Int J Mol Sci. 2026 Mar 11;27(6):2572. doi: 10.3390/ijms27062572 (PMC13026275; doi:10.3390/ijms27062572)
Supplement: Supplementary file 1 [file ijms-27-02572-s001.zip › ijms-4159605 File S5.pdf]

國泰醫院專題研究計畫執行同意書

立同意書人即本研究計畫主持人：**賴宗炫**，接受國泰醫院補助下述專題研究計畫：

計畫名稱：**利用生物資訊學探索子宮內膜異位症的分子機制與無創診斷標誌物-第一年研究**

計畫編號：CGH-MR- A11406

補助經費： 新台幣 **伍拾萬** 元整

茲願依國泰醫院有關規定執行本計畫，並同意遵守下列規定：

一、本計畫執行期間自民國 114年7月1日起至115年6月30日止，補助項目以國泰醫院審查通過之專題研究計畫經費核定清單所列為準。

二、本計畫之補助經費，於執行期滿前，依報支程序，檢據核實報銷。

三、本計畫（含多年期計畫全程計畫）執行期滿六個月內，須撰寫研究成果報告，辦理結案。並於2年內，發表論文於國泰醫院認可之peer-reviewed期刊。

四、本計畫之研發成果及其智慧財產權，須遵照"國泰綜合醫院專利權及著作權管理辦法"之規定。

五、本計畫執行中如涉及人體試驗或採集人體檢體，主持人應依有關法令規定辦理並檢具受試驗者或接受檢體採集者承諾同意書，受試（檢）者如為限制行為能力或無行為能力人，則應取得其法定代理人之書面同意書，並經執行機構核准，始得進行人體實驗或採集檢體。實驗過程應顧及人道並尊重受試（檢）者個人權益與安全措施，如發生人體實驗或採集檢體之法律問題，均由主持人自負完全責任；如有動物實驗，亦同意遵守有關法令暨本於愛護動物之態度進行；如有進行基因重組、具危害性微生物或病毒之實驗，應遵守相關法令規定並確實做好安全防護措施。

六、本計畫主持人對於計畫內容及研究成果涉及專利或其他智慧財產權者，應保證絕無侵害他人權利、違反醫藥衛生規範及影響公共秩序或善良風俗。其因而造成國泰醫院之權利或名譽受損者，國泰醫院得依法主張權利或追究其法律責任，並得要求損害賠償。

七、研發成果，其公開有影響民生福祉、國家安全、社會秩序或善良風俗之虞者，不宜公開。計畫主持人未經國泰醫院同意，擅自公開該研發成果，相關責任由計畫主持人自行負責。

八、計畫主持人如未依規定辦理經費結案，繳交研究成果報告及發表論文時，國泰醫院不再核給專題研究計畫之補助。

九、計畫主持人執行研究計畫應依科技資料保密要點及其他相關法令規定處理。

十、本同意書一式兩份，分由國泰醫院及計畫主持人收執，以資信守。

此致

國泰綜合醫院

計畫主持人：賴宗炫(簽名或蓋章)

執行單位或科別：婦女醫學部

中華民國 114年06月27日
